# Surgical Management of Pediatric Lymphatic Malformations: A Cross‐Sectional Study of Outcomes, Complications, and Recurrence at a Tertiary Care Center

**DOI:** 10.1002/hsr2.72084

**Published:** 2026-03-13

**Authors:** Nashwa Mohammed Al Nwaijie, Ahmad Alkheder, Adel Azar, Basem Alarsan Alyaseen, Abdulmajeed Yousfan

**Affiliations:** ^1^ Department of Pediatric Surgery Children University Hospital, Damascus University Damascus Syria; ^2^ Faculty of Medicine Aleppo University Aleppo Syria; ^3^ Department of Otorhinolaryngology Al‐Mouwasat University Hospital, Damascus University Damascus Syria; ^4^ Faculty of Medicine Damascus University Damascus Syria; ^5^ Faculty of Medicine Syrian Private University Damascus Syria; ^6^ Faculty of Dentistry Damascus University Damascus Syria

**Keywords:** children, complications, lymphatic malformations, pediatrics, recurrence, surgery

## Abstract

**Background and Aims:**

Lymphatic malformations (LMs) are congenital anomalies of the lymphatic system, frequently affecting pediatric populations. While complete surgical excision remains a primary therapeutic option, factors influencing outcomes, including lesion location and age at intervention, remain inadequately explored. This study investigates surgical outcomes, complications, and recurrence rates associated with LMs in children at a tertiary care center over a 6‐year period.

**Methods:**

A retrospective, cross‐sectional study was conducted at Children University Hospital (Damascus University) from April 2017 to May 2023. Forty children aged 0–12 years underwent surgical intervention for LMs. Data were collected on demographics, lesion location, age at intervention, surgical approaches, and postoperative outcomes. Associations between surgical comprehensiveness, age, lesion site, and complications were analyzed using descriptive statistics.

**Results:**

Head and neck were the most common lesion sites (55%), with 25% of cases operated on after 5 years of age. Complete excision was achieved in 82.5% of cases, with no recurrence in this group. Complications, including temporary nerve paresis and seromas, occurred in 25% of cases, predominantly in older children. Early intervention (6–12 months) yielded a 100% success rate with no complications or recurrences, suggesting this as an optimal window for surgery.

**Conclusions:**

Early surgical intervention for LMs, particularly within the first year of life, may offer favorable outcomes with minimal complications in carefully selected cases. While complete excision remains a goal when feasible, individualized management—including sclerotherapy or systemic therapy—should be considered based on lesion characteristics and multidisciplinary evaluation.

**Abbreviations**
LMs: Lymphatic Malformations
ICU: Intensive Care Unit
CT: Computed Tomography
MRI: Magnetic Resonance Imaging

## 1 Introduction

Lymphatic malformations (LMs), previously termed lymphangiomas, are congenital malformations of the lymphatic system, arising from abnormal development of lymphatic channels during embryogenesis, often posing significant diagnostic and therapeutic challenges [1]. These malformations are present in approximately 1 in 200 to 1 in 4000 live births [2]. Despite their prevalence, the optimal management strategies for LMs remain a subject of ongoing research. These lesions can present in a variety of forms, ranging from localized cystic hygromas to diffuse infiltrative malformations, affecting various anatomical sites, including the head and neck, mediastinum, and extremities, the head and neck area is the most common region [3]. The clinical presentation of LMs is highly variable, depending on the size, location, and extent of the malformation. Symptoms can include swelling, pain, disfigurement, and functional impairment, significantly impacting the patient's quality of life [4]. While several treatment modalities exist, including sclerotherapy, laser therapy, and surgical excision, complete surgical resection remains a cornerstone of management, particularly for localized lesions [5, 6]. However, Sclerotherapy is primarily employed as a treatment when surgical excision is challenging or not feasible, as it reduces the size of these cystic components [7].

However, achieving complete excision can be complex, especially in cases of extensive or infiltrative disease, and recurrence is a recognized complication [6, 8]. However, there remains limited data on the precise factors influencing surgical outcomes, particularly in pediatric populations. This cross‐sectional study was conducted at Children University Hospital to analyze the comprehensiveness of surgical excision in children with LMs and to assess the association between surgical outcomes, patient age at intervention, and lesion location. This study aims to provide valuable insights into the surgical management of pediatric LMs within our specific patient population.

## 2 Methods

A retrospective, cross‐sectional study was conducted at Children University Hospital (Damascus University). The study included all pediatric patients (0–12 years) with a confirmed LM who underwent surgical intervention. Patients with massive, diffuse infiltrative LMs deemed unresectable by multidisciplinary evaluation and primarily managed with non‐surgical therapies were excluded. We reviewed the medical records of all pediatric patients who underwent surgical intervention for lymphatic malformations between April 2017 and May 2023.

Children aged between 0 and 12 years old were included in this study. Preoperative assessments involve ultrasound and contrast‐enhanced imaging such as computed tomography (CT) or magnetic resonance imaging (MRI), chosen based on the lesion's location. Complete excision, partial excision, and partial excision with marsupialization were performed in all cases, either pre‐ or post‐sclerotherapy. Gender, age at surgical intervention, case presentation, location of the lesion, comprehensiveness of the excision, need for intensive care unit (ICU) and drainage placement were studied. Follow‐up occurred during these 6 years to detect recurrence and any disorders occurred thereafter. The aim of the study is to analyse the comprehensiveness of the excision according to the location of the lymphatic malformation and to assess complications with age‐related surgery.

### 2.1 Statistical Methodology

SPSS version 26 was used in this study. Description of the variables was occurred by means of frequencies and percent. Association between comprehensiveness of the excision with age at surgical intervention, location of the lesion and recurrence were inquired.

## 3 Results

40 patients with lymphatic malformations in different sites of the body were involved in this study. 22 cases located in the neck (55%) to be the most common site of these malformations with additional two cases (5%) extended into mediastinum. The largest proportion of cases aged at surgical intervention (25%) above 5 years (> 60 months) followed by 1–2 years (12–24 months) group (22.5%) and 2–5 years (24–60 months) group (17.5%). 85% of cases presented with mass only with two cases (5%) presented with infection and previous abscess formation in the neck and above the right femoral bone. lymphatic drainage is the typical course after excision which explains 50:50 of drain placement. Two cases recurred with partial and partial + marsupialization groups. Only two case indicated ICU entry. The first one was to a female 10 days old (case number 4 on Table 4) and the second case belong to a female 9 months old which had mediastinum lymphatic malformation impinged on right bronchi which opened when attempting complete excision of the lesion. A part of the wall of the lesion was left and bronchi sutured and ICU indicated for 2 days. Descriptive statistics are shown in (Table 1).

**Table 1:** Descriptive statistics of the study.

		Frequency	Percent
Gender	Male	19	47.50%
	Female	21	52.50%
Age	0–1 m	6	15%
	1–6 m	3	7.50%
	6–12 m	5	12.50%
	12–24 m	9	22.50%
	24–60 m	7	17.50%
	> 60 m	10	25.00%
Presentation	Mass	34	85%
	Infection + abscess	2	5%
	Increase in volume	1	2.50%
	Respiratory obstruction	1	2.50%
	Abdominal expansion	2	5%
Excision	Complete	33	82.50%
	Partial	1	2.50%
	Partial + marsupialization	6	15%
Location	Neck	22	55%
	Neck + mediastinum	2	5%
	Mediastinum	1	2.50%
	Body (axilla, thoracic wall, abdominal wall)	10	25%
	Internal organs (small intestinal, retroperitoneal)	4	10%
	Extremities	1	2.50%
Recurrence	Yes	2	5%
	No	38	95%
ICU	Yes	2	5%
	No	38	95%
Drainage	Yes	20	50%
	No	20	50%

Abbreviations: ICU, intensive care unit; m, month.

75% of cases have no complications and no wound infections. However, there were eight cases of seroma (4 in the neck, which is the most common location, and 4 in various other sites). Temporary facial and phrenic paresis occurred to a 16 months female had a neck lesion extended into the mediastinum and infiltrated with epicardium and superior and inferior vena cava. The paresis resolved spontaneously after 1 month for facial nerve and 5 months for phrenic nerve after surgery. Temporary accessory nerve paresis was seen in 11 years old female which has a massive neck lesion and resolved 1 week after surgery. 50% of complications occurred with 12–24 months group and 30% with 24–60 months group. So, there is increasing complications with delayed intervention after 1 year old. In addition, early intervention for infants can be indicated if a life‐threatening situation is entailed because organs are difficult to identify intraoperatively from the involved lymphoid areas. Moreover, complete excision was achieved in 100% of cases with 6–12 months group with no complications or recurrence are seen (Table 2).

**Table 2:** Relation between age and comprehensiveness of the excision and complications.

		Excision			Complications		Total
		Complete	Partial	Partial + marsupialization	Yes	No	
Age	0–1 m	4 (66.7%)	0	2 (33.3%)	1 (16.7%)	5 (83.3%)	6 (100%)
	1–6 m	2 (66.7%)	0	1 (33.3%)	0	3 (100%)	3 (100%)
	6–12 m	5 (100%)	0	0	0	5 (100%)	5 (100%)
	12–24 m	8 (88.9%)	0	1 (11.1%)	4 (44.4%)	5 (55.6%)	9 (100%)
	24–60 m	6 (85.7%)	0	1 (14.3%)	2 (28.6%)	5 (71.4%)	7 (100%)
	> 60 m	8 (80%)	1 (10%)	1 (10%)	3 (30%)	7 (70%)	10 (100%)
	Total	33 (82.5%)	1 (2.5%)	6 (15%)	10 (25%)	30 (75%)	40 (100%)

Abbreviation: m, month.

No recurrence with complete excision group was seen as opposed to cases operated with partial+ marsupialization group which recurred by 16.7% (1/6). Complete excision achieved in 33 cases (82.5%) across different sites of the body. One partial excision operated to 10‐year‐old female situated in the neck near the mandible and parotid gland, remnant of the lesion remained infiltrated within the parotid gland and this case was complicated with sialo fistula due to parotid duct injury and recurred after 6 months. A second intervention was indicated with no recurrence for 5 years (Table 3).

**Table 3:** Relation between comprehensiveness of the excision and location and recurrence.

		Excision			Total
		Complete	Partial	Partial + marsupialization	
Location	Neck	19 (86.4%)	1 (4.5%)	2 (9.1%)	22 (100%)
	Neck + mediastinum	1 (50%)	0	1 (50%)	2 (100%)
	Mediastinum	1 (100%)	0	0	1 (100%)
	Body	9 (90%)	0	1 (10%)	10 (100%)
	Internal organs	3 (75%)	0	1 (25%)	4 (100%)
	Extremities	0	0	1 (100%)	1 (100%)
Recurrence	Yes	0	1 (50%)	1 (50%)	2(100%)
	No	33 (86.8%)	0	5 (13.2%)	38 (100%)
	Total	33 (82.5%)	1 (2.5%)	6 (15%)	40 (100%)

Six cases (15%) intervened by partial excision and marsupialization and were as the following (Table 4).

**Table 4:** Partial excision and marsupialization cases.

Number	Gender	Age	Location	Recurrence	Notes
1	Male	2 m	Retroperitonial	No recurrence after 3 years.	
2	Male	1 y	Neck + mediastinum	No recurrence after 2 years	
3	Male	25 d	Above the femoral bone	No recurrence after 5 years.	
4	Female	10 d	Axilla	—	Brachial plexus infiltration, ICU indicated then died after 5 days due to respiratory weaning and choking by milk feeding.
5	Female	3 y	Neck	Recurred after 3 months	Carotid sheath infiltration
6	Female	6 y	Neck	No recurrence after 5 years	

Abbreviations: d, day; ICU, intensive care unit; m, month; y, year.

## 4 Discussion

The precise cause of lymphatic anomalies remains poorly understood, but research suggests they arise from errors during embryological development of the lymphatic system. In 1902, Sabin hypothesized that the lymphatic system originates from primordial endothelial buds, which sprout from the developing venous system around 6.5 weeks of gestation. These buds merge to form primitive plexuses, beginning in the cervical region. By approximately 7.5 weeks, caudal buds emerge and similarly develop into plexuses. At around 9.5 weeks, these plexuses begin to consolidate, and by 10 weeks, the central conducting lymphatics coalesce into continuous channels. Malformations are thought to result from defective primordial buds that fail to reconnect with their venous plexus of origin. Peripheral lymphatic malformations likely stem from the abnormal sequestration of proliferating lymphatic mesenchyme, which impairs the formation of a proper plexus network in peripheral regions of the body. Factors within the local tissue environment, such as density and composition, may influence the growth patterns of these malformations. In 1980, Van Der Putte provided additional evidence supporting Sabin's theory through detailed microscopic analysis of human embryos [4]. The management of these lesions is determined by several factors, including the clinical presentation, lesion size, anatomic location, and associated complications. Achieving complete excision is frequently challenging. While smaller lesions can typically be removed with favorable outcomes, larger lesions that extend into deeper regions such as the neck, tongue, and mediastinum carry a higher risk of complications. These may include the development of fistulas, infections, vascular damage, nerve injury, and cosmetic disfigurement. Historical reports indicate that mortality rates for such complex cases range from 2% to 6% [9, 10, 11]. Lymphatic malformations frequently develop in areas with a high density of lymphatic vessels. The head and neck region represent the most common site, comprising 45%–52% of cases. These malformations are also frequently observed in the axillary region, mediastinum, groin, and retroperitoneum, reflecting their predilection for lymphatic‐rich anatomical locations [12, 13, 14].

This cross‐sectional study provides valuable insights into the surgical management of lymphatic malformations (LMs) in a pediatric population at a single institution over a 6‐year period. Our findings highlight several key aspects of LM management, including lesion location, age at intervention, surgical approach, and postoperative outcomes.

The predominance of head and neck LMs (55%, with an additional 5% extending into the mediastinum) in our cohort aligns with existing literature, which consistently identifies this region as the most common site of involvement [15, 16]. This prevalence underscores the importance of expertise in managing complex anatomical structures in this area, particularly given the proximity to vital neurovascular structures. The varied clinical presentations observed, ranging from simple masses (85%) to more complex presentations involving infection (5%), respiratory obstruction (2.5%), and abdominal expansion (5%), emphasize the heterogeneous nature of these malformations and the need for individualized treatment strategies.

Our study revealed a distribution of patient ages at the time of surgical intervention, with the largest proportion (25%) being older than 5 years (> 60 months), followed by the 1–2 year (12–24 months) group (22.5%). This distribution suggests a trend towards delayed intervention in some cases. While the optimal timing of intervention remains a subject of debate, our findings, coupled with the analysis of complications in relation to age, suggest that delaying intervention beyond 1 year may be associated with an increased risk of complications. Specifically, 50% of complications occurred in the 12–24 month age group and 30% in the 24–60 month age group. This observation may be attributed to the progressive growth of LMs over time, leading to more extensive involvement of surrounding tissues and increased surgical complexity in older children. Conversely, early intervention in infants, particularly in life‐threatening situations involving airway compromise or vital organ compression, may be warranted despite the challenges of identifying anatomical structures in younger patients. The 100% complete excision rate with no complications or recurrence in the 6–12 month age group further supports the consideration of intervention within this timeframe when clinically appropriate. This finding suggests that a window of opportunity may exist within the first year of life where surgical intervention can be performed with optimal outcomes [17].

Complete excision was achieved in the majority of cases (82.5%), which is consistent with the goal of surgical management to achieve complete removal of the malformation. However, partial excision (2.5%) and partial excision combined with marsupialization (15%) were also performed in some cases, often dictated by the extent and location of the lesion. Notably, all recurrences (100%) observed in our study occurred in patients who underwent partial excision or partial excision with marsupialization, highlighting the importance of complete excision whenever feasible. The case of the 10‐year‐old female with a neck lesion near the mandible and parotid gland, who experienced recurrence and sialo fistula due to parotid duct injury after partial excision, further emphasizes the challenges associated with incomplete removal and the potential for significant complications. This case underscores the importance of meticulous surgical technique and careful dissection in complex anatomical locations to minimize the risk of iatrogenic injury.

The analysis of partial excision and marsupialization cases provided further insights. While four patients in this group experienced no recurrence after several years of follow‐up, indicating the potential utility of this approach in select cases, the other two cases highlighted potential limitations and risks. One patient experienced recurrence after 3 months, one had brachial plexus infiltration and sadly succumbed to postoperative complications. This variability in outcomes underscores the importance of careful patient selection for partial excision and marsupialization and the need for close postoperative monitoring. The unfortunate case of the 10‐day‐old female who succumbed to postoperative complications highlights the inherent risks associated with surgical intervention in very young infants, especially in cases with extensive disease and involvement of critical structures.

Our findings demonstrate that early surgical intervention, particularly within the 6–12 month age range, is associated with superior outcomes, including a 100% complete excision rate and absence of complications or recurrence. This suggests a critical window where anatomical clarity and lesion containment may facilitate safer dissection. Conversely, delayed presentation beyond 1 year correlated with increased complication rates, likely due to lesion expansion and infiltration into complex anatomical planes. The overall low recurrence rate following complete excision (0%) underscores its curative potential when achievable, while partial procedures carried higher recurrence risks, necessitating careful patient selection. These results emphasize that timely, radical resection—when feasible—should be prioritized in appropriately selected cases to minimize morbidity. In resource‐limited settings, where advanced adjunct therapies may be scarce, early surgical referral remains a cornerstone of management. These findings advocate for a proactive surgical approach within the first year of life, supported by multidisciplinary evaluation and tailored intervention strategies.

The low rate of ICU admission (5%) and the equal distribution of drainage placement (50%) suggest that the majority of procedures were well‐tolerated. The occurrence of temporary facial, phrenic, and accessory nerve paresis in a few cases highlights the potential for nerve injury during surgery, particularly in cases involving extensive neck lesions. However, the spontaneous resolution of these pareses in all cases is reassuring and emphasizes the importance of conservative management in these situations. The seroma formation in eight cases (20%) is a known complication of LM surgery and was managed conservatively.

Postoperative complications encompass facial and hypoglossal nerve injuries, seroma formation, tissue defects, bleeding, infections, Horner's syndrome, and vesicle development at the incision site. In our study, the incidence of surgical complications closely aligned with those reported in existing literature. Specifically, complication rates for surgeries targeting lesions in the cervicofacial region, chest, and extremities have been documented to range between 12% and 33%, which is consistent with our findings [18, 19, 20, 21].

As clinical Recommendations: 1) This study highlights the advantages of intervening during infancy (6–12 months), where the likelihood of achieving complete excision without complications is highest. Early referral to specialized centers is crucial for optimizing outcomes. 2) Advanced imaging and multidisciplinary planning are essential, particularly for complex cases involving deep‐seated or infiltrative lesions. 3) While complete excision remains the gold standard, partial excision with or without marsupialization may be necessary for cases involving critical structures. In such cases, close follow‐up is essential to monitor for recurrence. 4) Patients with complications, particularly those involving nerve injuries or ICU admission, require close monitoring and rehabilitation to ensure recovery.

AbouZeid et al. (2023) similarly advocate early intervention to minimize complications. While their cohort (*n* = 131) emphasized sclerotherapy and sirolimus for complex cases, our surgical‐focused analysis (*n* = 40) underscores the feasibility of complete excision in infants (6–12 months), mirroring their observation that delayed management increases procedural risks. Both studies highlight the head/neck predilection of LMs and the role of individualized approaches [17].

Surgical intervention remains a primary treatment option for well‐localized lymphatic malformations; however, its role becomes more limited in cases of extensive, infiltrative, or complex disease. For such challenging scenarios, systemic therapy with oral sirolimus has emerged as a valuable alternative. The prospective multicenter trial by Ji et al. (2021) demonstrated its significant efficacy in reducing lesion volume and improving quality of life in pediatric patients with complicated vascular anomalies, including those with a prominent lymphatic component. This supports the use of sirolimus either as a neoadjuvant therapy to facilitate subsequent resection or as a primary treatment for unresectable, diffuse LMs. Integrating sirolimus into a multidisciplinary treatment algorithm is crucial for optimizing outcomes when complete surgical excision is not feasible [22].

This study provides valuable data on the surgical management of pediatric LMs, particularly in a resource‐limited setting. However, our study has some limitations. Some data points were unavailable in this retrospective study, single‐center study, which may limit the generalizability of our findings to broader populations and could have constrained the comprehensiveness of our analyses. Specifically, [malformation type, intralesion bleomycin injection, detailed surgical approach, and decision‐making for surgical intervention] were not available. The relatively small sample size may limit the statistical power to detect subtle associations. Additionally, the cross‐sectional design does not allow for the establishment of causal relationships. Future prospective, multi‐center studies with larger sample sizes and longer follow‐up periods are needed to further validate our findings and to explore the long‐term outcomes of surgical management for pediatric LMs.

## 5 Conclusion

This study supports complete surgical excision as the goal of management for pediatric lymphatic malformations when feasible. Our experience suggests that intervention within the first year of life may be associated with improved outcomes, including higher rates of complete excision and fewer complications. Delayed intervention appears linked to increased surgical complexity and higher complication rates, likely due to lesion expansion and infiltration. Individualized management, including partial procedures when necessary, remains important. Further prospective studies are needed to confirm these associations and refine timing strategies.

## Author Contributions

**Nashwa Mohammed Al Nwaijie:** writing – original draft; formal analysis; validation; investigation; conceptualization; methodology; software; data curation; visualization; writing – review and editing. **Ahmad Alkheder:** Writing – review and editing; writing – original draft; resources; visualization; funding acquisition; formal analysis; validation; investigation; software; methodology; conceptualization. **Adel Azar:** Conceptualization; methodology; software; validation; formal analysis; visualization; writing – review and editing; writing – original draft. **Basem Alarsan Alyaseen:** Writing – review and editing; writing – original draft; visualization; validation; data curation; methodology. **Abdulmajeed Yousfan:** Writing – review and editing; writing – original draft; project administration; resources; visualization; validation; supervision.

## Funding

The authors received no specific funding for this work.

## Ethics Statement

Damascus University's ethics approval committee examined and authorized this study (number 155,2023). The ethical approval was conducted according to the principles of the Declaration of Helsinki. This study does not require participant consent, as it is a retrospective study based on archival records, and our institution does not require patients' consent to use their records as long as they are anonymous data.

## Conflicts of Interest

The authors declare no conflicts of interest.

## Transparency Statement

The lead author Ahmad Alkheder affirms that this manuscript is an honest, accurate, and transparent account of the study being reported; that no important aspects of the study have been omitted; and that any discrepancies from the study as planned (and, if relevant, registered) have been explained.

## Data Availability

The data that support the findings of this study are available from the corresponding author upon reasonable request. All data generated or analyzed during this study are included in this article and can be requested from the corresponding author Ahmad Alkheder. Ahmad Alkheder had full access to all of the data in this study and takes complete responsibility for the integrity of the data and the accuracy of the data analysis. All authors have read and approved the final version of the manuscript.

## References

[1] M. Wassef , F. Blei , D. Adams , et al., “Vascular Anomalies Classification: Recommendations From the International Society for the Study of Vascular Anomalies,” Pediatrics 136, no. 1 (2015): e203–e214, 10.1542/peds.2014-3673. 26055853

[2] M. Poget , M. Fresa , O. El Ezzi , G. Saliou , M. T. Doan , and A. de Buys Roessingh, , “Lymphatic Malformations in Children: Retrospective Review of Surgical and Interventional Management,” Pediatric Surgery International 39, no. 1 (2022): 36. 10.1007/s00383-022-05320-x. 36469112 PMC9722885

[3] J. B. Mulliken and J. Glowacki , “Hemangiomas and Vascular Malformations in Infants and Children: A Classification Based on Endothelial Characteristics,” Plastic and Reconstructive Surgery 69, no. 3 (1982): 412–420, 10.1097/00006534-198203000-00002. 7063565

[4] R. G. Elluru , K. Balakrishnan , and H. M. Padua , “Lymphatic Malformations: Diagnosis and Management,” Seminars in Pediatric Surgery 23, no. 4 (2014): 178–185, 10.1053/j.sempedsurg.2014.07.002. 25241095

[5] B. B. Lee , Y. W. Kim , J. M. Seo , et al., “Current Concepts in Lymphatic Malformation,” Vascular and Endovascular Surgery 39, no. 1 (2005): 67–81, 10.1177/153857440503900107. 15696250

[6] J. Yan , Y. Fu , S. Liu , Y. Bai , and Y. Chen , “Mesenteric and Omental Lymphatic Malformations in Children: Seven‐Year Surgical Experience From Two Centers in China,” BMC Pediatrics 24, no. 1 (2024): 360. 10.1186/s12887-024-04808-w. 38783260 PMC11112925

[7] S. Wiegand , B. Eivazi , A. P. Zimmermann , A. M. Sesterhenn , and J. A. Werner , “Sclerotherapy of Lymphangiomas of the Head and Neck,” Head & Neck 33, no. 11 (2011): 1649–1655, 10.1002/hed.21552. 20737487

[8] J. Chiara , G. Kinney , J. Slimp , G. S. Lee , S. Oliaei , and J. A. Perkins , “Facial Nerve Mapping and Monitoring in Lymphatic Malformation Surgery,” International Journal of Pediatric Otorhinolaryngology 73, no. 10 (2009): 1348–1352, 10.1016/j.ijporl.2009.06.008. 19592118

[9] J. A. Perkins , S. C. Manning , R. M. Tempero , et al., “Lymphatic Malformations: Review of Current Treatment,” Otolaryngology–Head and Neck Surgery 142, no. 6 (2010): 795–803, 10.1016/j.otohns.2010.02.026. 20493348

[10] L. J. Fliegelman , D. Friedland , M. Brandwein , and M. Rothschild , “Lymphatic Malformation: Predictive Factors for Recurrence,” Otolaryngology–Head and Neck Surgery 123, no. 6 (2000): 706–710, 10.1067/mhn.2000.110963. 11112962

[11] G. C. Kang and C. Song , “Forty‐one Cervicofacial Vascular Anomalies and Their Surgical Treatment—Retrospection and Review,” Annals of the Academy of Medicine, Singapore 37, no. 3 (2008): 165–179. 18392293

[12] M. Hochman , D. M. Adams , and T. D. Reeves , “Current Knowledge and Management of Vascular Anomalies, II: Malformations,” Archives of Facial Plastic Surgery 13, no. 6 (2011): 425–433, 10.1001/archfacial.2011.795. 22106189

[13] R. G. Elluru and R. G. Azizkhan , “Cervicofacial Vascular Anomalies. II. Vascular Malformations,” Seminars in Pediatric Surgery 15, no. 2 (2006): 133–139, 10.1053/j.sempedsurg.2006.02.011. 16616317

[14] L. M. de Serres , K. C. Y. Sie , and M. A. Richardson , “Lymphatic Malformations of the Head and Neck. A Proposal for Staging,” Archives of Otolaryngology–Head and Neck Surgery 121, no. 5 (1995): 577–582, 10.1001/archotol.1995.01890050065012. 7727093

[15] A. H. Hassanein , J. B. Mulliken , S. J. Fishman , N. A. Quatrano , D. Zurakowski , and A. K. Greene , “Lymphatic Malformation: Risk of Progression During Childhood and Adolescence,” Journal of Craniofacial Surgery 23, no. 1 (2012): 149–152, 10.1097/SCS.0b013e3182413ea8. 22337394

[16] G. Tasnádi , “Epidemiology and Etiology of Congenital Vascular Malformations,” Seminars in Vascular Surgery 6, no. 4 (1993): 200–203. 8305974

[17] A. A. AbouZeid , S. A. Mohammad , N. H. Aly , et al., “Lymphatic Malformations: A 9‐year Experience at the Vascular Anomaly Clinic,” Egyptian Pediatric Association Gazette 71, no. 1 (2023): 88. 10.1186/s43054-023-00236-0.

[18] P. E. Burrows , R. K. Mitri , A. Alomari , et al., “Percutaneous Sclerotherapy of Lymphatic Malformations With Doxycycline,” Lymphatic Research and Biology 6, no. 3–4 (2008): 209–216, 10.1089/lrb.2008.1004. 19093794

[19] S. J. Boardman , L. A. Cochrane , D. Roebuck , M. J. Elliott , and B. E. J. Hartley , “Multimodality Treatment of Pediatric Lymphatic Malformations of the Head and Neck Using Surgery and Sclerotherapy,” Archives of Otolaryngology–Head & Neck Surgery 136, no. 3 (2010): 270–276, 10.1001/archoto.2010.6. 20231646

[20] T. Okazaki , S. Iwatani , T. Yanai , et al., “Treatment of Lymphangioma in Children: Our Experience of 128 Cases,” Journal of Pediatric Surgery 42, no. 2 (2007): 386–389, 10.1016/j.jpedsurg.2006.10.012. 17270554

[21] J. C. Moreno‐Alfonso , P. Triana , M. Miguel Ferrero , M. Díaz González , and J. C. López Gutiérrez , “Risk Factors for Sequelae After Surgery for Lymphatic Malformations in Children,” Journal of Vascular Surgery. Venous and Lymphatic Disorders 12, no. 2 (2024): 101730, 10.1016/j.jvsv.2023.101730. 38070670 PMC11523444

[22] Y. Ji , S. Chen , K. Yang , et al., “A Prospective Multicenter Study of Sirolimus for Complicated Vascular Anomalies,” Journal of Vascular Surgery 74, no. 5 (2021): 1673–1681.e3, 10.1016/j.jvs.2021.04.071. 34082006

